# Local recurrence at the site of the Lone Star device through implantation of exfoliated cells during local excision for early rectal cancer: A case report

**DOI:** 10.1016/j.ijscr.2022.106891

**Published:** 2022-03-01

**Authors:** A.S. van Lieshout, A.A.J. Grüter, L.J.H. Smits, P.J. Tanis, J.B. Tuynman

**Affiliations:** a Department of Surgery, Amsterdam UMC, Vrije Universiteit, Cancer Center Amsterdam, the Netherlands; b Department of Surgical Oncology and Gastrointestinal Surgery, Erasmus MC, Rotterdam, the Netherlands

**Keywords:** Rectal cancer, Anal metastasis, Lone star retractor, TAMIS, Local recurrence, Implantation of exfoliated cells

## Abstract

**Introduction:**

Invasive procedures for colorectal cancer can cause iatrogenic tumor cell seeding. Implantation of these exfoliated cells in the surrounding tissue can result in locoregional cancer recurrence. This has been described in endoscopic procedures and major surgical resections, however recurrence in iatrogenic lesions of the anal canal during minimal invasive rectal surgery has not been shown in literature yet. This is the first reported case of recurrent rectal cancer that developed into an anal metastasis at the site where hooks of the Lone Star Retractor disrupted the epithelial lining of the anal canal during a local excision of early rectal cancer using TAMIS.

**Presentation of case:**

A 57 year old male was diagnosed with a high risk early stage rectal adenocarcinoma. He was treated with transanal minimally invasive surgery (TAMIS) with the use of a Lone Star retractor and he received subsequent chemo-radiotherapy. 23 months later the patient developed a bleeding mass bulging out of the anus. A true cut and incision biopsy was performed and the pathology report revealed localization of adenocarcinoma at the anal canal which was similar to the earlier diagnosed rectal carcinoma. The patient underwent an abdominal perineal resection and left-sided lymph node dissection.

**Discussion and conclusion:**

This shows that local recurrence through implantation of exfoliated tumor cells can occur in iatrogenic lesions of the anal canal not only in major but also in minimal invasive rectal surgery. Careful tissue handling and rectal washout may reduce the chance of this implantation metastasis.

## Highlights

• Recurrent rectal cancer can occur at the site of an iatrogenic anal lesion.
• Anal metastasis can develop due to implantation of exfoliated rectal tumor cells.
• Iatrogenic local recurrence occurs in both major rectal surgery and local excision.
• Careful tissue handling and rectal washout may reduce the chance of recurrence.

## 1 Introduction and importance

Invasive procedures for colorectal cancer can cause iatrogenic tumor cell seeding [1]. Implantation of these exfoliated cells in the surrounding tissue can result in locoregional cancer recurrence [2]. This has been shown for both endoscopic procedures and surgical resections for colorectal cancer [3], [4], [5], [6]. It is thought that free intraluminal cancer cells during endoscopy implant in mucosal or serosal lesions of the intestinal tract and may lead to locoregional ent-metastases [7]. For transanal minimally invasive surgery (TAMIS) procedures in patients with early rectal cancer, endoluminal recurrences have been described. However recurrence in iatrogenic lesions of the anal canal during TAMIS is not yet described in literature. We present the first reported case of recurrent rectal cancer that developed into an anal metastasis at the site where hooks of the Lone Star Retractor disrupted the epithelial lining of the anal canal during a local excision of early rectal cancer using TAMIS. This case report has been reported in line with the SCARE 2020 criteria [8].

## 2 Presentation of case

**Fig. 1:**
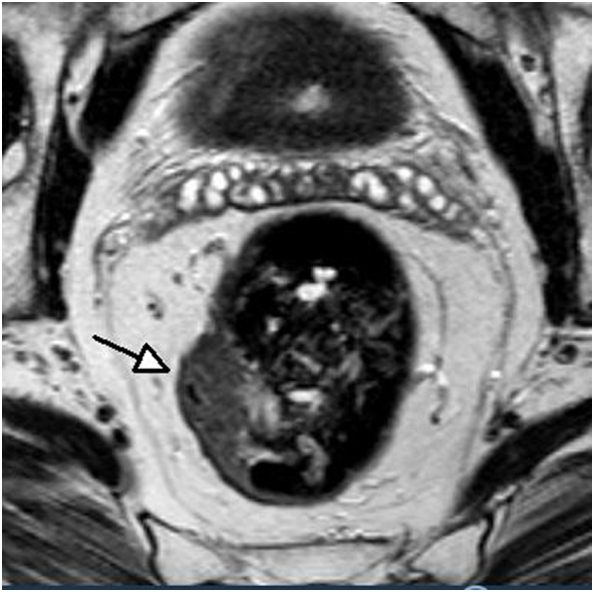
MRI scan at first presentation shows a tumor mass of 2 cm located 11 cm proximal to the anal verge at the right side of the rectum.

A 57 year old male was presented with anal blood loss, intermittent diarrhea, rectal tenesmus and a palpable mass in the rectum. Laboratory results showed a normal carcinoembryonic antigen (CEA) level of <5 μg/L. Colonoscopy with polypectomy of nine polyps and biopsies of a malignant lesion in the rectum was performed. Pathology examination revealed a well differentiated adenocarcinoma of the mid rectum. Abdominal MRI showed a lesion with a diameter of 2 cm located 8 cm proximal to the anorectal junction without infiltration of the muscularis propria and no suspicious lymph nodes or extramural vascular invasion (EMVI) [Fig. 1]. On the additional CT imaging of chest and abdomen, no signs of distant metastases were observed, resulting in cT1N0M0 stage according to the American Joint Committee on Cancer (AJCC) classification. Since the tumor was a small T1 rectal cancer without signs of suspicious lymph nodes, a local excision was proposed as rectal preserving therapy. This was done through TAMIS with Lone Star retraction of the anal canal and a Gelpoint platform. Rectal washout with povidone iodine was performed following the local excision. The pathology report showed a complete resection of a stage pT1 tumor according to the Union for International Cancer Control (UICC) classification with free resection margins, however vascular invasion was seen and therefore complementary treatment was indicated.

In this case, the patient participated in a randomized controlled trial that compares completion total mesorectal excision (TME) versus adjuvant chemo radiotherapy after local excision in patients with intermediate risk early rectal cancer (TESAR trial) [9]. He was randomized to the chemo-radiotherapy arm and received small field radiation of the rectum and mesorectum (25 × 1.8 Gy) with concomitant capecitabine (825 mg/m^2^ twice a day) for 5 weeks. Strict follow-up evaluation and imaging after 6, 12 and 18 months did not show any signs of recurrence.

**Fig. 2:**
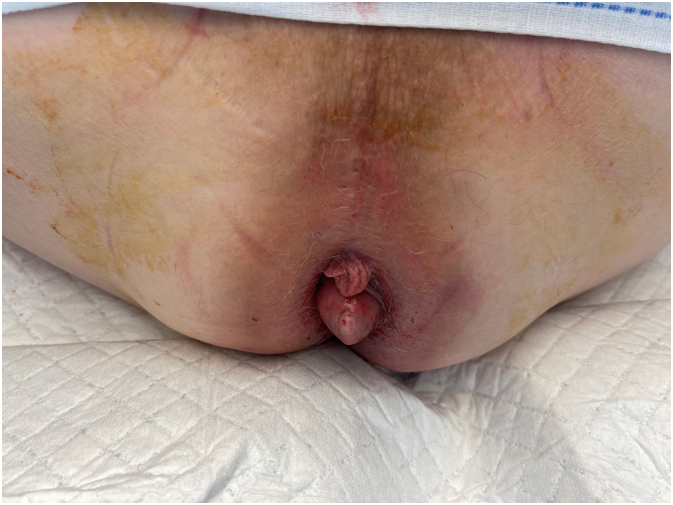
Photo of the tumor recurrence during physical examination 23 months after TAMIS: bleeding, painless mass bulging out of the anus.

**Fig. 3:**
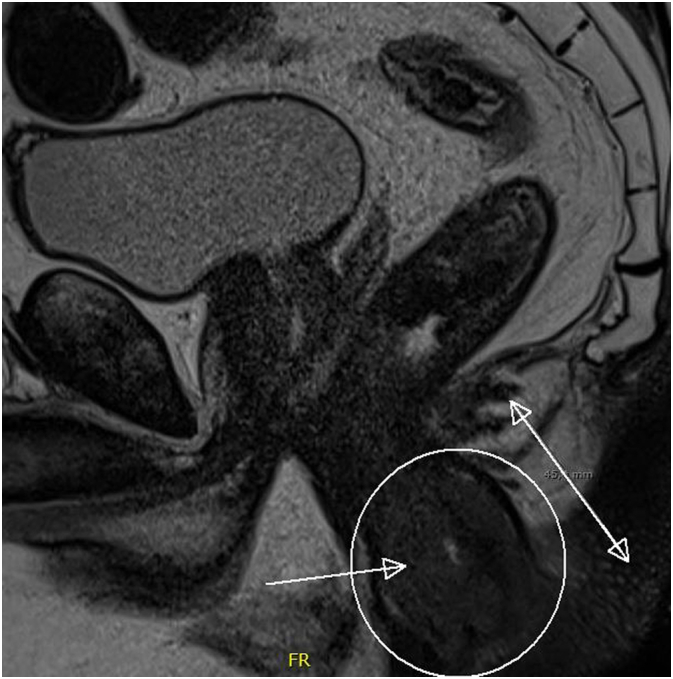
MRI scan at recurrence shows a mass in the anal canal bulging out of the anus.

**Fig. 4:**
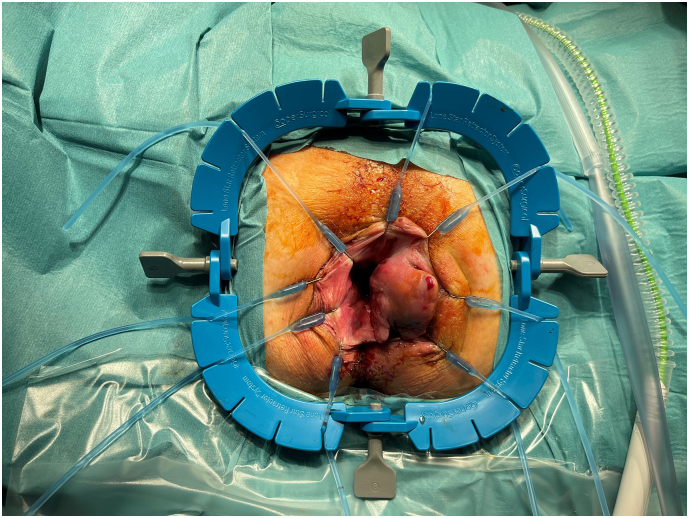
Photo of the tumor recurrence during inspection at the operation room. A true cut and incision biopsy was taken.

However, after 23 months the patient developed a painless, growing and bleeding mass bulging out of the anus [Fig. 2]. The differential diagnosis included malignancy (recurrence or anal cancer) and a thrombosed hemorrhoid. Imaging showed involvement of the internal sphincter and intersphincteric space and a left-sided suspicious inguinal lymph node of 19 mm [Fig. 3]. Inspection at the operating room was performed. The mass appeared to be 4 cm located at the site of the Lone star Retractor used during the TAMIS procedure and was suspected for a submucosal malignancy [Fig. 4]. A true cut and incision biopsy of the mass and biopsy of the lymph node was performed since en-bloc resection was not possible. The pathology report revealed localization of adenocarcinoma at the anal canal and inguinal node which was similar to the earlier diagnosed rectal carcinoma. The TAMIS scar was free of tumor. Additional imaging showed no distant metastases.

The patient underwent an abdominal perineal resection and left-sided lymph node dissection. Pathology showed stage rpT2N1b with radical removal and 22 resected lymph nodes of which 2 were positive for metastasis. Postoperative recovery was complicated by wound infection for which vacuum sponge and antibiotic treatment was initiated. Furthermore, the patient had an uneventful recovery. The patient is currently at two years follow up after TAMIS and approximately one year after APR surgery. No new recurrence was seen and no further adjuvant treatment was given.

## 3 Clinical discussion

Spreading of rectal tumor cells to the anal canal or perianal region is rare. Colorectal cancer usually metastasizes via hematogenous route to the liver, lungs, bone, nervous system, via lymphatics to regional and distant nodes, and with intraabdominal exfoliated cancers cells to the peritoneum [10], [11]. However several cases of perianal metastasis have been described [2]. These metastases are thought to be a result of three possible mechanisms: (1) implantation of exfoliated intraluminal tumor cells in preexisting lesions of the anal canal such as fissuras [12], fistulas [2], [13], [14], [15], [16], [17] and anal crypts [17], (2) lymphovascular metastasis [7], and (3) seeding and implantation of tumor cells into injured anal tissue caused by an invasive procedure [7]. Both anal metastases at the site of preexisting lesions and hematogenous or lymphatic metastases have well been described in literature although the latter occurs less frequently [2]. However, implantation of rectal tumor cells in the perianal region during an invasive procedure is scarce. This type of metastasis has been described with the use of a circular stapler [5], [6], [18], [19], a perianal purse string suture [20], [21], a Gelpi retractor [22], [23], a hemorrhoidectomy scar [24], [25] and a Lone Star retractor [26], [27].

The Lone Star retractor is a frequently used flexible and adjustable self-retaining retractor system designed to maximize visualization during anorectal surgery. It is placed at the anal verge by using small hooks penetrating the mucosa of the anal canal resulting in small scars. In this case, the recurrence was located in the anal canal below the dentate line close to the normal epithelium of the skin where the Lone Star hooks were located during TAMIS. Based on the anatomy and embryological origin of the rectum it is unlikely that the recurrence in the anal canal was due to vascular invasion and therefore we presume that tumor cell implantation occurred in one of the Lone Star lesions. So far only three cases of Lone Star metastasis have been reported of which all of them in the context of anterior resection [26], [27]. This is the first case of an anal iatrogenic metastasis after local excision, which is a less invasive procedure with a probably lower change of tumor spill. It is important to draw attention to the phenomenon of implantation of malignant cells in both major and minimally invasive rectal cancer surgery.

To prevent implantation metastases, the rectum and anal canal are washed with cytotoxic solutions such as povidone‑iodine. Despite the available conflicting data and lack of randomized clinical trials, rectal washout is recommended to be routinely performed in rectal surgery until more convincing evidence is obtained [28], [29], [30], [31], [32]. In this case, rectal washout was performed following local excision, however it did not prevent exfoliating cells from attaching to the Lone Star site. Surgeons should be careful with tissue handling and try to minimize traumatic use of instruments.

In this case the patient participated in a clinical trial and was randomized in the experimental arm. The patient received adjuvant chemoradiation instead of standard completion surgery. The chemoradiation field is limited to the rectum and mesorectum, therefore the implanted tumor cells in the anal canal did not receive any of this treatment. Completion surgery might have prevented this if an abdominoperineal resection would have been performed, but not in case of sphincter preserving surgery.

## 4 Conclusion

We present a rare case of anal implantation metastasis at the site of a scar form a Lone Star Retractor hook that developed following local excision using TAMIS approach. This shows that this phenomenon can occur not only in major but also in minimal invasive rectal surgery. Careful tissue handling and rectal washout may reduce the chance of implantation metastasis during invasive procedures for rectal cancer.

## Sources of funding

This research did not receive any specific grant from funding agencies in the public, commercial, or not-for-profit sectors.

## Ethical approval

Waived by ethical committee of our institute.

## Consent

Written informed consent was obtained from the patient for publication of this case report and accompanying images. A copy of the written consent is available for review by the Editor-in-Chief of this journal on request.

## Author contribution

A.S. van Lieshout, A.A.J. Grüter and L.J.H. Smits wrote the paper. J.B. Tuynman and P.J. Tanis revised and edited the paper. J.B. Tuynman and P.J. Tanis performed the surgical procedure.

## Registration of research studies

This work is registered in Research Registry with the unique identifying number: researchregistry7527.

## Guarantor

Ass. prof. dr. J.B. Tuynman.

## Provenance and peer review

Not commissioned, externally peer-reviewed.

## Declaration of competing interest

All authors have no conflict of interest.
